# Virulence and Antimicrobial Resistance in *Campylobacter jejuni* and *Campylobacter coli*

**DOI:** 10.3390/antibiotics15090830

**Published:** 2026-08-26

**Authors:** Minchae Kang, Jinkyung Lee, Hyokeun Song

**Affiliations:** 1 Department of Pharmacy, Wonkwang University, Iksan-si 54538, Jeollabuk-do, Republic of Korea; 2 Institute of Pharmaceutical Research and Development, College of Pharmacy, Wonkwang University, Iksan-si 54538, Jeollabuk-do, Republic of Korea

**Keywords:** *Campylobacter jejuni*, *Campylobacter coli*, virulence, cytolethal distending toxin, lipooligosaccharide, antimicrobial resistance

## Abstract

*Campylobacter jejuni* and *Campylobacter coli* account for most recognized cases of human campylobacteriosis. For *C. coli*, gene-carriage surveys often stand in for direct tests of function; this distinction is maintained throughout the review. Virulence is discussed as a sequence of events rather than as a catalogue of factors. Motility and adhesion have the most reproducible support during colonization and epithelial contact, whereas biofilm formation, quorum sensing, and T6SS-associated phenotypes vary substantially across strains and models. We then consider host–cell injury, persistence, transmission, and the uncommon post-infectious outcomes linked to sialylated LOS. Antimicrobial resistance is treated in parallel, with emphasis on target-site change, ribosomal protection or methylation, enzymatic inactivation, and CmeABC-mediated efflux. The One Health model places both species within overlapping animal, food, and environmental reservoirs rather than assigning *C. jejuni* exclusively to poultry or *C. coli* to swine. A narrower conclusion emerges from the resistance-fitness literature: *gyrA* Thr-86-Ile and CmeABC can alter colonization or competitive fitness, but the available data do not show that resistance generally predicts more severe human disease. This distinction frames the proposed priorities for surveillance and intervention.

## 1. Introduction

*Campylobacter jejuni* and *Campylobacter coli* are thermotolerant, Gram-negative, microaerophilic, curved bacteria. Together, these species account for most cases of human campylobacteriosis [1]. Both species colonize the intestinal tract of poultry and other food-producing animals, providing multiple routes of exposure through undercooked poultry, cross-contaminated foods, unpasteurised milk, and untreated water. In many surveillance systems, campylobacteriosis ranks among the most frequently reported bacterial enteric infections and contributes substantially to the foodborne disease burden [2,3].

Human disease is usually acute gastroenteritis. Diarrhea can be watery or bloody, and fever and abdominal pain are common. Most patients improve within a week. The exceptions matter clinically: prolonged or systemic infection occurs more often in young children, older adults, and immunocompromised patients [4]. Post-infectious complications are much less frequent and include Guillain-Barré syndrome (GBS), reactive arthritis, and post-infectious irritable bowel syndrome [5]. *C. jejuni* accounts for most diagnosed infections; *C. coli* is recovered less often from patients but tends to carry more antimicrobial resistance.

**Figure 1:**
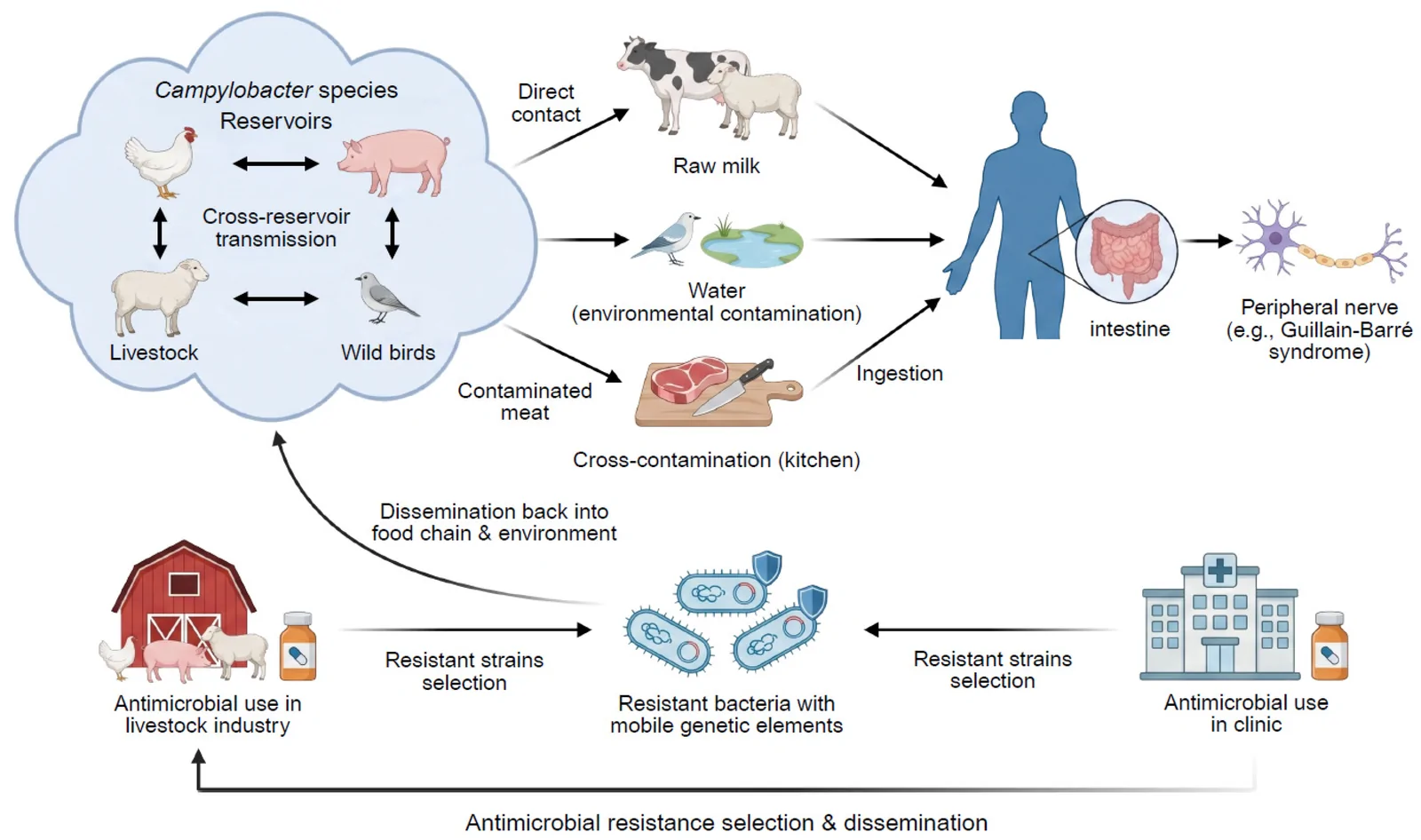
One Health transmission routes of *Campylobacter* and pathways for antimicrobial resistance dissemination. Food animals, wild birds, water, and environmental sources are shown as connected rather than isolated reservoirs. Human exposure can occur through foodborne routes, raw or unpasteurized milk, contaminated water, kitchen cross-contamination, or direct animal contact. Antimicrobial use in livestock and clinical care provides selection pressure, and resistant strains or mobile elements may re-enter food and environmental pathways. Created in BioRender. Song, H. (2026) https://BioRender.com/72vcvx9, accessed on 21 July 2026.

*Campylobacter* is important not only because it causes enteritis but also because the same populations circulate through food animals, food products, people, and the environment. Virulence depends on a collection of traits rather than a single determinant, including motility, attachment, toxin activity, and persistence [5,6]. Resistance adds a distinct clinical pressure. Fluoroquinolone resistance is already common in many settings, while macrolide resistance, though often lower, affects a major treatment option. These trends are tracked by national and international surveillance programs [4,7,8,9]. The broader antimicrobial resistance (AMR) burden provides context: bacterial resistance was associated with an estimated 1.27 million deaths in 2019 [10]. For *Campylobacter*, the relevant question is therefore not simply how virulence and resistance coexist, but whether specific resistance mechanisms also change fitness at the human–animal-environment interface (Figure 1).

Recent reviews have examined *Campylobacter* virulence [5,6] and antimicrobial resistance [11] extensively. The present study adopts a narrower focus by assigning each virulence factor to the stage of infection for which the strongest experimental evidence is available, then assessing how this evidence varies across models and species. The present study applies the same distinction in the antimicrobial-resistance section, particularly when fitness is inferred from colonization or competition rather than from disease severity. This approach is especially important for *C. coli*, for which gene detection is considerably more common than direct functional validation.

## 2. Literature Search Strategy

We searched PubMed/MEDLINE, Web of Science, and Scopus from the inception of the databases. The literature search was updated during revision, with the final update completed on 15 August 2026. Because this review aimed to provide a narrative synthesis rather than a systematic review, the search was used to identify mechanistic studies, surveillance datasets, and authoritative clinical guidance. Virulence-related searches combined *Campylobacter*, *C. jejuni*, or *C. coli* with terms related to flagella, chemotaxis, adhesion, CadF, FlpA, Cia, HtrA, cytolethal distending toxin, lipooligosaccharide, capsule, biofilm, quorum sensing, type VI secretion, and Guillain-Barré syndrome. Resistance-related searches included fluoroquinolones/gyrA; macrolides/23S rRNA/erm(B); tetracycline/tet(O); beta-lactams/blaOXA-61; aminoglycosides; CmeABC; multidrug-resistance genomic islands; fitness; colonization; and clonal lineage. WHO, CDC/NARMS, EFSA/ECDC, and European Commission sources were consulted when a claim depended on surveillance or current clinical guidance. Evidence was assessed primarily according to what each study design could establish. For mechanistic claims, direct experimental evidence from defined genetic perturbation was weighted more heavily than gene carriage alone, particularly when the phenotype was confirmed in vivo. Cell-culture experiments were used to characterize cellular effects, whereas genomic and epidemiological studies primarily informed distribution and association. For the evidence-strength categories used in the virulence summary, “Strong” denotes direct experimental support, preferably involving a defined mutant and in vivo confirmation; “Moderate” denotes experimental support with limited in vivo validation; and “Context-dependent” denotes findings that vary according to strain, reservoir, host, or experimental model. Gene carriage or epidemiological association without functional testing was not interpreted as evidence of causality. These labels are qualitative narrative judgments and are not equivalent to a formal GRADE assessment.

## 3. Epidemiology and Clinical Burden

Any global estimate of campylobacteriosis is approximate because many infections are not tested, and diagnostic practice continues to change. Nevertheless, available estimates indicate a substantial burden. In 2010, WHO’s Foodborne Disease Burden Epidemiology Reference Group attributed approximately 96 million foodborne illnesses to *Campylobacter* [2,3]. Diarrheal diseases accounted for the largest proportion of the estimated 33 million foodborne disability-adjusted life-years (DALYs). One DALY represents one year of healthy life lost to premature death or disability [12]. Analyses restricted to animal-source foods reached a similar conclusion, placing *Campylobacter* alongside nontyphoidal *Salmonella* among the leading contributors to foodborne DALYs [13]. The burden is particularly high on young children in low- and middle-income countries, where repeated early-life infections have also been associated with environmental enteropathy [14]. In high-income surveillance systems, *Campylobacter* is often the most frequently reported bacterial cause of enteritis; however, its global rank depends on testing practices, surveillance coverage, and the pathogens included in the comparison.

Most infections remain gastrointestinal. Watery diarrhea is common, but some patients develop inflammatory or bloody diarrhea with fever and abdominal cramping. Recovery within a week is typical. Bacteremia and other prolonged or systemic manifestations are uncommon overall and are seen more often in young children, older adults, and immunocompromised patients [4]. Among the recognized post-infectious complications, GBS carries the greatest clinical consequence; *C. jejuni* is the bacterial antecedent identified most often [15,16].

The percentages in Table 1 should be read as snapshots of defined study populations, not as regional prevalence estimates. Human clinical isolates, retail-food isolates, and animal isolates are sampled differently, and resistance estimates can shift with study period, susceptibility method, and breakpoint. For that reason, Table 1 retains source and testing information beside each percentage. Comparable standardized monitoring is still sparse across much of Africa, Latin America, South Asia, and Southeast Asia.

Table 1 is limited to ciprofloxacin, erythromycin, and tetracycline because these drugs are reported consistently across both clinical and food-animal *Campylobacter* datasets and correspond to the resistance mechanisms emphasized here. The cited percentages apply only to the populations from which they were measured. EU harmonized monitoring also includes chloramphenicol, ertapenem, and gentamicin under Commission Implementing Decision (EU) 2020/1729. Cross-study comparisons therefore need to account for species, isolate source, sampling period, testing method, and interpretive criteria.

## 4. Clinical Management and Treatment Implications

For uncomplicated campylobacteriosis, rehydration and other supportive measures are usually sufficient. Antibiotics are reserved for patients with severe or prolonged illness, systemic infection, or an increased risk of complications, including young children, older adults, pregnant patients, and immunocompromised patients [4]. When antimicrobial therapy is indicated, a macrolide, usually azithromycin, is generally preferred. Empirical ciprofloxacin may be unreliable in settings with high fluoroquinolone resistance [17,22]. In severe invasive infection, an aminoglycoside or carbapenem may be considered, particularly when multidrug resistance or a non-jejuni/non-coli species is involved. Species identification, susceptibility testing, and current local guidance are most useful in these situations [11].

Current CDC guidance is consistent with this approach: antibiotics are not required for most infections, but azithromycin is commonly used when treatment is indicated. Where fluoroquinolone resistance is prevalent, ciprofloxacin should not be assumed active without local resistance data or susceptibility results [22].

## 5. Virulence Mechanisms

**Figure 2:**
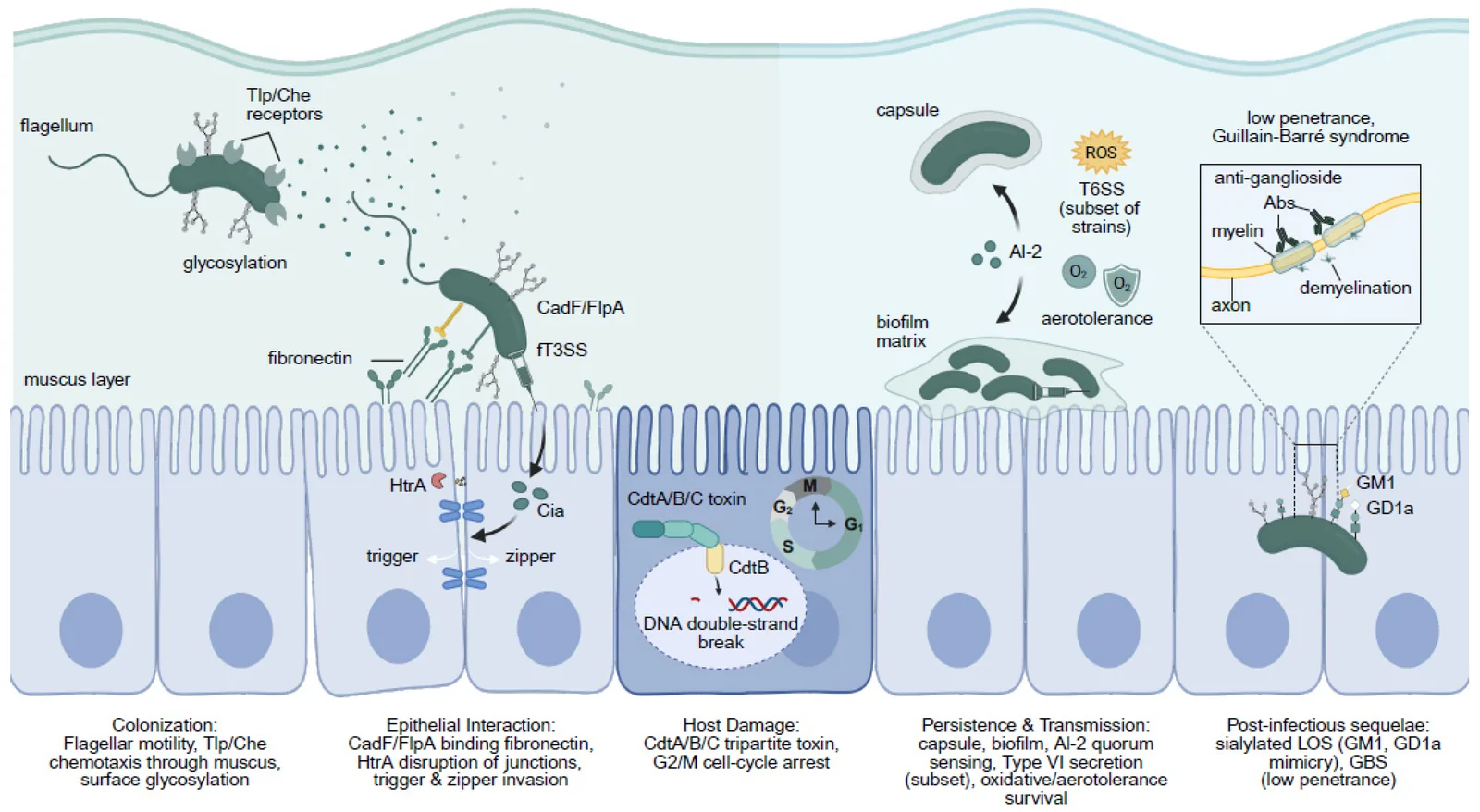
Virulence mechanisms of *C. jejuni* across infection stages. Motility, chemotaxis, surface glycosylation, and CadF/FlpA-mediated attachment dominate the early colonization and epithelial-contact stages. Cia secretion uses the flagellar export apparatus, while HtrA and strain-dependent invasion contribute to epithelial interaction. CDT is shown separately as the best-defined toxin-mediated mechanism of host–cell damage. Capsule variation, biofilm-associated growth, quorum sensing, T6SS in selected strains, and stress responses are grouped with persistence or transmission. Sialylated LOS is placed at the post-infectious stage because ganglioside mimicry precedes GBS only in a small subset of infections. Created in BioRender. Song, H. (2026) https://BioRender.com/72vcvx9, accessed on 21 July 2026. Abbreviations: fT3SS, flagellar type III secretion system; T6SS, type VI secretion system; CDT, cytolethal distending toxin; LOS, lipooligosaccharide; GBS, Guillain-Barré syndrome.

*Campylobacter* lacks the classical non-flagellar type III secretion system found in many enteric pathogens. Instead, its pathogenic processes involve multiple stages, beginning with movement through the intestinal niche and epithelial attachment, then extending to tissue injury, persistence, and, rarely, post-infectious autoimmunity [5,6,23]. Table 2 and Figure 2 follow this sequence. We interpret evidence for invasion and intracellular persistence cautiously because these phenotypes vary according to strain, host, and experimental model and should not be considered universal characteristics of *Campylobacter*.

The evidence base is substantially imbalanced between species. Most direct virulence studies have examined *C. jejuni*, whereas *C. coli* is more frequently represented in PCR-based surveys, comparative-genomic studies, or analyses that infer function from conserved homologues. Accordingly, Table 2 identifies the species and principal experimental model associated with each factor and indicates when *C. coli* has been functionally tested. The absence of such experiments is treated as an evidence gap rather than as evidence that the mechanism is absent.

### 5.1. Colonization: Motility, Chemotaxis, and Surface Glycosylation

Flagellar motility is one of the most reproducible colonization phenotypes reported for *Campylobacter*. Aflagellate and non-motile mutants colonize chicks poorly, and *flaA* also contributes to epithelial-cell invasion in vitro [24,25,26]. Chemotaxis is supported by a different set of experiments: Tlp and Che mutants lose the ability to navigate efficiently toward favorable mucus-associated niches, which compromises colonization [5,6]. Those data do not establish a comparable effect on human disease severity. Surface glycosylation broadens the picture further. The *pgl*-dependent N-glycosylation pathway and extensive O-glycosylation alter surface and secreted proteins, and mutant studies link these changes to attachment, colonization, and immune interaction [27].

### 5.2. Epithelial Interaction and Host–Cell Damage

CadF and FlpA anchor the evidence for epithelial attachment, although the two adhesins are not supported by identical experiments. FlpA is required for efficient chicken colonization; CadF has been tested functionally in both *C. jejuni* and *C. coli* [28,29]. Campylobacter also uses its flagellar export apparatus to secrete Cia proteins through the flagellar type III secretion system (fT3SS). A ciaB mutant secretes less Cia protein and invades cultured cells less efficiently, and secretion requires an intact flagellum [30,31]. Evidence for invasion itself is less uniform. Cell-culture and selected in vivo studies describe membrane ruffling and adhesin-driven entry, but neither route appears obligatory across strains [41]. HtrA provides another route to epithelial disruption by cleaving junctional proteins and facilitating paracellular passage; it also contributes to bacterial stress tolerance [32]. Thus, epithelial interaction is well supported, whereas the importance of any single invasion route remains model- and strain-dependent [5].

CDT provides the clearest toxin mechanism in *Campylobacter*. CdtA and CdtC contribute to binding and delivery, whereas CdtB is the DNase-active subunit. Once delivered, CdtB causes DNA damage, arrests host cells at G2/M, and can produce cell distension and death [33]. These cellular effects are reproducible. Their contribution to the severity of naturally acquired human disease is much harder to quantify, which is why mechanistic confidence is stronger than disease-causality confidence in Table 2. Outer membrane vesicles offer one plausible route for CDT delivery [34].

### 5.3. Persistence, Surface Variation, and Transmission

Persistence and transmission depend on a less uniform set of traits than early attachment. The phase-variable capsule contributes to serum resistance and surface variation [1]. Biofilm growth and AI-2-associated signaling can improve survival under environmental or food-processing conditions, but most supporting experiments are in vitro, and *luxS* phenotypes differ among strains. LuxS also participates in the activated methyl cycle, so a *luxS*-dependent phenotype is not automatically evidence of quorum sensing [35]. This distinction is relevant to intervention studies; for example, decanoic and lauric acids inhibit AI-2-associated signaling and biofilm formation in *C. jejuni* [42]. T6SS is even more variable, occurring only in a subset of isolates and with prevalence that changes by source, geography, and detection method [36]. Where it is present, studies have linked it to oxidative-stress tolerance, host–cell interaction, colonization, and interbacterial competition. Other persistence mechanisms are less dramatic but ecologically important. Outer membrane vesicles can deliver toxins and proteases [34], whereas aerotolerance, oxidative-stress responses, and entry into a viable but nonculturable state help cells survive processing and environmental exposure [37,38]. These traits can increase opportunities for transmission without necessarily increasing human disease severity [6].

Phase variation adds another source of heterogeneity. LOS, capsule, and flagellar glycosylation can switch through slipped-strand mispairing at homopolymeric contingency loci. Because those changes are rapid and reversible, cells within the same lineage can differ in surface recognition, neuropathy-associated LOS structures, and colonization phenotype [1].

### 5.4. Post-Infectious Sequelae: LOS Ganglioside Mimicry and Guillain-Barré Syndrome

The LOS outer core of *C. jejuni* can acquire phase-variable sialylated structures that mimic human gangliosides such as GM1 and GD1a. Such mimicry can elicit cross-reactive autoantibodies capable of injuring peripheral nerves. Sensitization with *C. jejuni* LOS produces anti-GM1 IgG and flaccid paralysis, and biosynthetic genes such as *cstII* and *cgtB* determine the relevant structures [15,16,43]. The pathway is nevertheless non-deterministic. Only a minority of strains carry neuropathy-associated sialylated LOS classes. Classes A, B, and C contain sialyltransferases, and class A is over-represented in GBS [40]. Sialylation is also associated with severe enteritis and reactive arthritis. It is not strictly required for invasive infections, suggesting pleiotropic effects [44]. Only a small fraction of *Campylobacter* infections progress to GBS, with approximately 0.2–1.7 cases per 1000 illnesses. The outcome depends on host susceptibility, the anti-ganglioside antibody repertoire, and complement activation. Different gangliosides are linked to different subtypes of Miller Fisher syndrome (MFS), such as GQ1b. The mechanistic link is therefore strong, but its clinical penetrance is low.

Reactive arthritis and post-infectious IBS also follow some *Campylobacter* infections, but neither has a bacterial trigger defined as clearly as the LOS-GBS pathway. For GBS, ganglioside mimicry provides a direct mechanistic bridge from a bacterial surface structure to host autoimmunity. The corresponding bacterial determinants for reactive arthritis and post-infectious IBS are less certain, and host susceptibility probably contributes more strongly [5,14].

### 5.5. Host Inflammatory Response

The avian-human contrast makes host context impossible to ignore. *Campylobacter* can colonize the avian intestine at high abundance with little overt pathology, yet the same species may cause a strongly neutrophilic enteritis in people. In epithelial models, bacterial products activate NF-κB-dependent IL-8 secretion and disrupt barrier integrity; CDT has also been implicated in this response [32,45]. *C. jejuni* flagellin is a poor TLR5 agonist because sequence changes alter the canonical recognition region, allowing motility with reduced TLR5 activation [46]. Intracellular *C. jejuni* can activate NLRP3 rather than NLRC4 and trigger caspase-1-dependent IL-1β release [47]. These pathways are well demonstrated experimentally, but their relative contribution to disease severity in patients is not resolved. Differences in innate recognition, together with temperature-adapted bacterial physiology, may help explain why avian colonization and human disease look so different [6].

## 6. Antimicrobial Resistance Profiles

**Figure 3:**
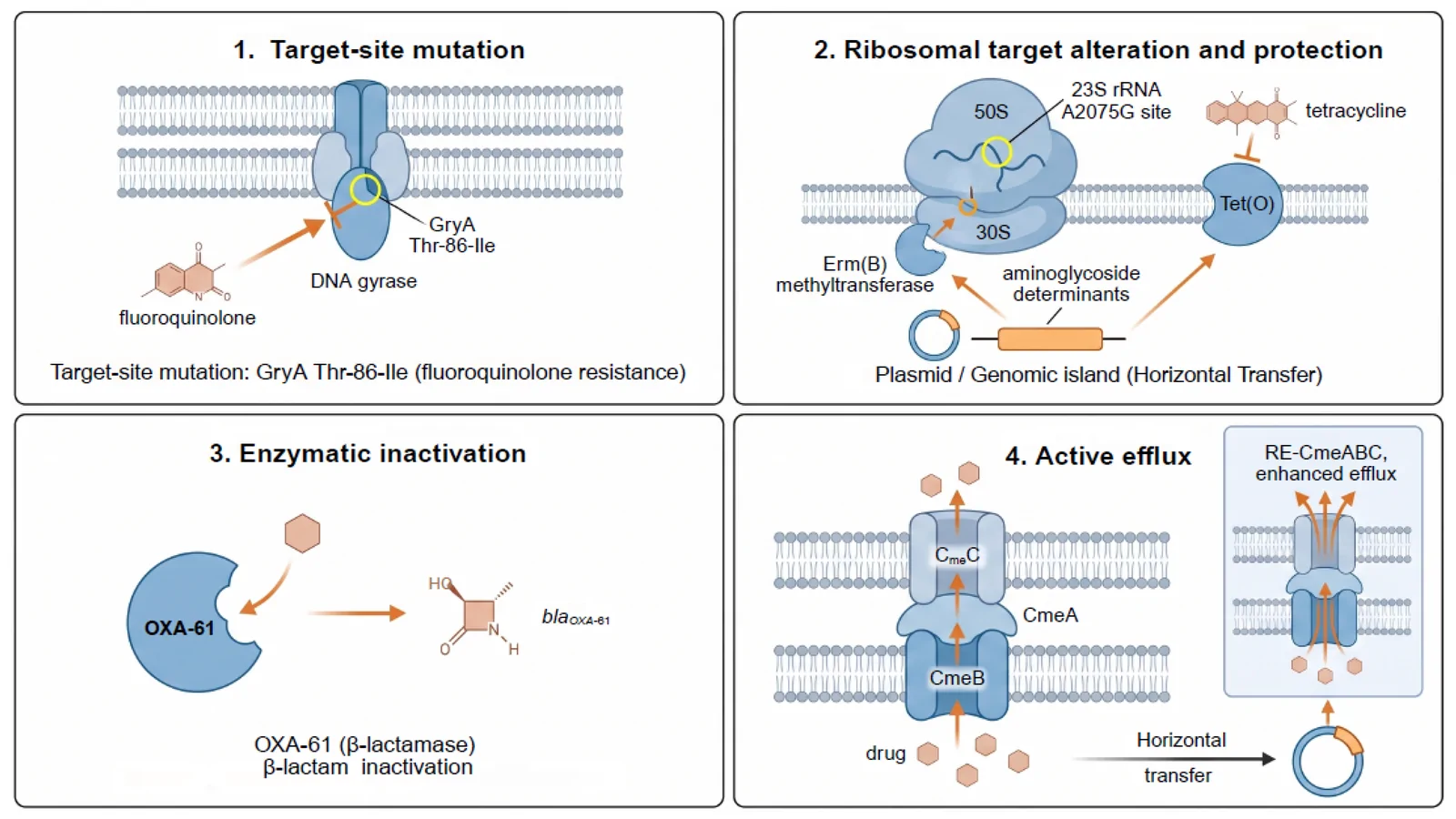
Major antimicrobial-resistance mechanisms in *Campylobacter*. The schematic links representative determinants to four mechanisms: *gyrA* Thr-86-Ile to fluoroquinolone target alteration; 23S rRNA A2075G, *erm(B)*, and *tet(O)* to ribosomal alteration or protection; *blaOXA-61* to beta-lactam inactivation; and CmeABC/RE-CmeABC to active efflux. Plasmids and MDRGIs indicate that several determinants, including *erm(B)*, *tet(O)*, and aminoglycoside-resistance genes, can move horizontally. Created in BioRender. Song, H. (2026) https://BioRender.com/72vcvx9, accessed on 21 July 2026. Abbreviations: RE-CmeABC, resistance-enhancing CmeABC; MDRGI, multidrug-resistance genomic island.

*Campylobacter* can reach the same resistant phenotype by several routes. Target alteration, target protection or modification, drug-inactivating enzymes, and active efflux may occur together in one strain and can reinforce one another [4]. Figure 3 summarizes the main examples discussed below.

### 6.1. Fluoroquinolone Resistance

DNA gyrase is the relevant fluoroquinolone target in *Campylobacter*. High-level resistance is most commonly associated with the *gyrA* Thr-86-Ile substitution (C257T) in the quinolone resistance-determining region [48]. The mutation can be identified directly with molecular assays, including droplet digital PCR [49]. Under fluoroquinolone exposure, resistant mutants are readily selected in vivo, and CmeABC contributes to this selection [50]. This resistance mechanism is unusual because its phenotype extends beyond MIC: Thr-86-Ile changes DNA supercoiling, and some resistant strains show greater in vivo competitiveness or biofilm formation [51,52,53]. Those fitness effects are examined in Section 7.

### 6.2. Macrolide Resistance

Macrolides act at the 50S ribosomal subunit and remain the preferred treatment when antimicrobial therapy for campylobacteriosis is required [4,17]. In *Campylobacter*, high-level resistance usually reflects mutations in 23S rRNA; A2075G is the dominant change, whereas A2074C/G is encountered less often. CmeABC can help stabilize the resistant phenotype during stepwise selection [54]. A distinct route appeared with the report of the transferable methylase gene *erm(B)* in 2014. *erm(B)* often sits within MDRGIs that carry resistance determinants for several drug classes, creating a platform for co-selection. To date, its dissemination has been documented, particularly in *C. coli* from China, and reflects both clonal expansion and horizontal transfer [55,56].

### 6.3. Tetracycline Resistance

Tetracycline resistance is mediated chiefly by the ribosomal protection protein Tet(O), encoded by *tet(O)*. The gene is frequently plasmid-borne and horizontally transferable, although efflux can also contribute [11]. Resistance is common among animal-derived isolates, although prevalence varies substantially by region (Table 1) [17].

### 6.4. Beta-Lactam Resistance

Beta-lactam resistance is common, with the chromosomal class D beta-lactamase OXA-61, encoded by *blaOXA-61* (Cj0299), providing one important mechanism. A single promoter nucleotide change can shift *blaOXA-61* from little or no expression to an expressed state, helping to explain why carriage of the gene does not always correspond to phenotypic resistance [57]. *blaOXA-61* does not account for all beta-lactam resistance. Low outer membrane permeability, CmeABC-mediated efflux, and other intrinsic factors also reduce susceptibility to beta-lactams [11]. These combined intrinsic mechanisms limit the therapeutic role of most beta-lactams, although carbapenems may retain activity in systemic infection.

### 6.5. Aminoglycoside Resistance and Multidrug-Resistance Genomic Islands

Gentamicin and other aminoglycosides remain relevant to selected systemic infections, with resistance historically reported at relatively low levels [17]. Resistance, when present, is commonly associated with aminoglycoside-modifying enzyme genes from the *aph*, *aac*, and *ant* families. Their frequent location within MDRGIs carrying *erm(B)* and other resistance determinants creates opportunities for coselection and horizontal dissemination across clinically relevant drug classes [56].

### 6.6. Multidrug Efflux: CmeABC and the RE-CmeABC Variant

CmeABC links drug resistance to a normal colonization function. The complex consists of the inner-membrane transporter CmeB, the periplasmic adaptor CmeA, and the outer-membrane channel CmeC. It exports structurally unrelated antimicrobials but also protects the bacterium from bile, a function required for efficient intestinal colonization [58,59]. CmeR represses the system, and target-site mutations often act with CmeABC to produce clinically relevant resistance. RE-CmeABC is a higher-activity variant with changes in the CmeB drug-binding pocket; these changes broaden efflux, raise fluoroquinolone resistance, and widen the ciprofloxacin mutant-selection window. The variant can be transferred horizontally. Clinical *C. jejuni* isolates carrying RE-CmeABC have been described, but current datasets are insufficient to define its population prevalence or temporal trend [60].

### 6.7. Species and Reservoir Differences: C. jejuni Versus C. coli

*C. jejuni* dominates human campylobacteriosis and is frequently linked to poultry, but cattle, sheep, wild birds, water, and other environmental sources also contribute. *C. coli* makes up a smaller and geographically variable fraction of human cases and is encountered often in swine [21]. Resistance is a more consistent point of difference than virulence: comparative animal studies usually report higher multidrug and macrolide resistance in *C. coli*, together with more frequent *erm(B)*/MDRGI carriage [55,56]. The two species nevertheless rely on much the same core mechanisms, including *gyrA* Thr-86-Ile, 23S rRNA A2075G, *tet(O)*, *blaOXA* alleles, and CmeABC. Species differences therefore reflect how often these determinants and mobile elements occur, not entirely different resistance biology [11].

Recent European primary studies broaden this reservoir perspective beyond broiler chickens. In Germany, Beterams et al. tracked Campylobacter contamination during processing in three broiler and one turkey slaughterhouses; counts generally declined during processing, but re- and cross-contamination remained evident at individual steps [61]. In Romania, Campylobacter from slaughtered turkeys showed high resistance to ciprofloxacin and tetracycline in both *C. jejuni* and *C. coli*, together with frequent detection of resistance and virulence-associated determinants [18]. A separate Romanian slaughterhouse study found *C. coli* in 92.3% of pig cecal samples and substantial resistance to tetracycline and ciprofloxacin, while *C. jejuni* was also recovered from cattle [19]. Turkey-farm MLST data from Italy further indicated that environmental and water sources accounted for most source-attributed isolates, followed by pests and breeders [62]. Campylobacter recovered from Romanian slaughterhouse and retail poultry meat also included ciprofloxacin- and tetracycline-resistant isolates, linking the production chain with food exposure [20]. Together, these studies support a One Health model in which poultry, swine, ruminants, food-processing environments, water, and other farm-associated sources overlap rather than forming exclusive species-reservoir pairs.

Virulence is the part of the species comparison where the evidence is least balanced. CadF is a useful exception because its function has been tested directly in both *C. jejuni* and *C. coli* [29]. For many other determinants, *C. coli* data stop at gene detection. Local PCR surveys have reported *flaA*, cadF, *ciaB*, and cdt, often with lower frequencies for the cdt cluster, but those surveys cannot establish either function or global prevalence [63]. FlpA homologues, capsule and glycosylation loci, and CmeABC are widely distributed across *Campylobacter* [5,11], yet comparable functional experiments in *C. coli* remain scarce, particularly for invasion, toxin delivery, and immune interaction. *C. coli* nevertheless causes a clinically similar gastroenteritis and accounts for a smaller, country-dependent fraction of diagnosed cases [14]. It is also reported less often before GBS develops [40]. Neither observation demonstrates lower intrinsic pathogenicity; exposure, diagnostic practice, and study intensity differ between the species. At present, resistance ecology is much better characterized than virulence biology for *C. coli*.

### 6.8. Unresolved Questions in Resistance

Several resistance determinants are well defined at the molecular level but still leave population-level questions unanswered. Macrolide resistance illustrates the problem. In many *C. jejuni* populations, it remains uncommon despite antimicrobial selection. A fitness cost from mutations across multiple 23S rRNA operons is one possible explanation, but exposure patterns, ecological niche, lineage structure, and access to *erm(B)*-bearing MDRGIs could generate the same observation [55]. *C. coli* shows the opposite pattern, with greater carriage of *erm(B)*-containing islands, yet existing studies do not clearly separate clonal expansion from ecological opportunity [56]. RE-CmeABC raises a similar uncertainty: current reports cannot distinguish true global expansion from preferential detection in selected datasets [60]. Genotype-phenotype prediction is also incomplete for beta-lactams and aminoglycosides because promoter activity and expression can alter susceptibility [57]. A different problem comes from culture-independent diagnostics, which reduce the number of isolates available for phenotypic testing and may gradually weaken long-term resistance surveillance [14].

## 7. Interplay Between Resistance and Fitness

Resistance, fitness, and virulence are related in some *Campylobacter* studies, but represent distinct biological outcomes. Here, fitness primarily refers to competition or persistence within a host or reservoir. Biofilm formation is considered a surface-associated persistence phenotype; host–cell pathogenicity refers to adhesion, invasion, or cytotoxicity; and clinical virulence refers to disease severity in people. These categories matter because a determinant can improve colonization or competition without making disease more severe. The evidence for *gyrA* and CmeABC is interpreted on that basis.

Among the available examples, the *gyrA* Thr-86-Ile substitution is particularly informative because its fitness effect has been tested in controlled competition experiments. Fluoroquinolone-resistant *C. jejuni* carrying the substitution outcompeted susceptible bacteria in chickens even without antibiotic exposure [51]. The effect mapped to the *gyrA* allele rather than to an obvious compensatory change. It was not universal, however: the same allele imposed a fitness cost in some genetic backgrounds. That result makes the biological interpretation more interesting than a simple “resistance is beneficial” model. Thr-86-Ile changes DNA supercoiling and can alter gene expression [52]; hence, its consequence depends on the lineage and resistance phenotype. CmeABC links resistance and fitness for a different reason. It is a conserved system, not an acquired resistance mutation, and its normal functions include both antimicrobial efflux and bile resistance required for intestinal colonization [58,59]. Selection for higher CmeABC activity can therefore change susceptibility while acting on a system already involved in colonization.

Mobile genetic elements create a more indirect connection between resistance and fitness. Notably, *erm(B)* and aminoglycoside-modifying enzyme genes can occur within the same MDRGI, allowing exposure to one antimicrobial to co-select multiple resistance determinants at once [55,56]. If a given island also carries genes that affect stress tolerance or colonization, those traits could travel with the resistance cassette. At present, however, that inference rests largely on co-location. Functional evidence that the mobile element itself improves fitness is still sparse.

Clonal expansion can produce the same appearance of coupling without a mechanistic link. Resistant isolates often belong to successful multilocus sequence-type complexes, and those lineages may carry virulence-associated traits for reasons unrelated to the resistance determinant [4,51]. A lineage that transmits well can therefore increase in frequency while bringing a resistance allele with it. In epidemiological datasets, this lineage effect can look like a resistance-fitness association even when the two traits are inherited independently.

Genetic background is the main obstacle to separating these possibilities. Fluoroquinolone resistance is unusually informative because isogenic mutants have been tested alongside in vivo competition assays. Equivalent evidence across several lineages is still rare for efflux variants, biofilm phenotypes, and mobile resistance elements. Simply adding more resistant isolates will not solve the problem. More informative studies would introduce the same determinant into multiple backgrounds, measure competition and colonization directly, and then connect those phenotypes with longitudinal genomic data. Fitness, biofilm formation, host–cell effects, and clinical severity should remain separate endpoints. With that standard, direct resistance-fitness coupling is best supported for *gyrA* and for the dual colonization/resistance role of CmeABC; most other proposed links remain associative. Strategies aimed at weakening both resistance and fitness through CmeABC or DNA-supercoiling targets are still preclinical. The resistance-fitness relationships and their evidence strengths are summarized in Table 3.

## 8. Discussion

The virulence literature is not equally persuasive for every factor, and that unevenness is biologically informative. Motility, chemotaxis, CadF, and FlpA repeatedly affect colonization, while CDT has a well-defined cellular mechanism. LOS ganglioside mimicry also has strong mechanistic support, although progression to GBS is rare. Biofilm formation, quorum sensing, and T6SS occupy a different evidentiary category: their phenotypes vary across strains and experimental systems, and direct links to human disease are less secure [35,36,42]. Host context adds another layer. *Campylobacter* can be tolerated in the avian gut yet drive inflammatory disease in people [6], so a virulence factor cannot be interpreted independently of the host in which it is measured.

Resistance presents a different problem. The molecular basis of fluoroquinolone and macrolide resistance is comparatively mature, but the newer uncertainties are about distribution and spread, particularly for aminoglycoside resistance, *erm(B)*-bearing MDRGIs, and RE-CmeABC [55,56,60]. Cross-study comparison remains difficult because surveillance datasets differ in geography, source, sampling period, and breakpoint definition, and many are only cross-sectional snapshots (Table 1). Harmonized genomic surveillance would improve comparability [17,64]. Species-level reporting is equally important: *C. coli* contributes fewer diagnosed human infections than *C. jejuni* but often carries more resistance, a contrast that disappears when results are collapsed to the genus level [21].

Resistance can also alter *Campylobacter* ecology in ways that an MIC value does not capture. Some fluoroquinolone-resistant genotypes persist after direct drug pressure is removed [51], indicating that their competitive behavior is not explained by susceptibility alone. The pattern is not general, however. Outside the *gyrA* and CmeABC examples, many reported resistance-fitness associations can still be explained by lineage background or other correlated traits [4].

For this reason, better colonization, stronger in-host competition, more biofilm, or altered host–cell phenotypes are not treated here as synonyms for clinical virulence. A claim of increased clinical virulence requires evidence tied to disease severity in the natural host.

Mechanistic studies and surveillance datasets also answer different questions. Laboratory experiments can isolate one pathway but often rely on a few reference strains. Surveillance captures population breadth, yet matched phenotypes, host metadata, and disease-severity data are frequently absent. The two evidence streams therefore connect only partially. This review has the same structural limitation. A narrative format allows mechanistic and surveillance evidence to be discussed together, but it does not provide the exhaustive retrieval or formal risk-of-bias assessment of a systematic review. The literature is also concentrated in well-studied reference strains, high-income surveillance systems, and English-language publications. Those skews matter when comparing the apparent importance of individual determinants.

## 9. Conclusions and Future Directions

The available evidence does not support a single pathogenic program for *Campylobacter*. Colonization, epithelial interaction, persistence, and host responses represent partly distinct traits, and the strength of evidence behind them differs. Antimicrobial resistance is similarly heterogeneous. Target changes, ribosomal protection or methylation, drug-inactivating enzymes, and efflux occur in different combinations, sometimes within the same strain. The strongest resistance-fitness links are narrower than the phrase “resistance-virulence coupling” suggests. Notably, *gyrA* Thr-86-Ile can change in-host competitiveness, and CmeABC contributes both to antimicrobial efflux and to intestinal colonization.

Whole-genome surveillance is most informative when sequence data are interpreted alongside phenotypic susceptibility, source, and lineage information. For *Campylobacter*, that means following *gyrA* Thr-86-Ile, 23S rRNA A2075G, *erm(B)*-associated MDRGIs, *tet(O)*, *blaOXA* alleles, CmeABC/RE-CmeABC variation, and LOS biosynthesis classes across well-defined isolate collections. No single reservoir provides the whole picture. Human, poultry, swine, cattle, food, water, and environmental isolates each represent different points in the transmission network. Comparable breakpoints and shared surveillance endpoints would make those collections easier to compare across regions. Poultry control remains important, and experimental poultry vaccination strategies may reduce *C. jejuni* colonization and onward transmission [65], but gaps in standardized data from underrepresented regions are equally consequential. Linking microbiological and genomic results with clinical and veterinary metadata is likely to yield more than expanding any one dataset in isolation.

A One Health interpretation also avoids two common shortcuts. Poultry is not synonymous with broilers, and neither do Campylobacter species belong to a single exclusive reservoir. Broilers and turkeys, swine, cattle, and other ruminants, wild birds, food products, water, and environmental pathways overlap. Surveillance that retains species and source information is therefore more informative than genus-level summaries or single-reservoir models [18,19,20,61,62,64].

## Figures and Tables

**Table 1:** Selected source-specific examples of antimicrobial resistance prevalence in *Campylobacter.*

Setting/Study Period	Species and Isolate Source	Ciprofloxacin	Erythromycin	Tetracycline	Testing/Interpretation
Republic of Korea, 2017–2021	*C. jejuni*; human diarrhea isolates	84.6%	0.6%	26.2%	Broth microdilution; CLSI/WHONET [17]
Romania, 2025	*C. jejuni*/*C. coli*; turkey cecal isolates	93.3%/85.7%	18.7%/28.6%	77.3%/93.7%	Broth microdilution; EU/EUCAST framework [18]
Romania, 2021	*C. coli*; pig cecal isolates	69.4%	8.3%	75.0%	Broth microdilution; EURL/EU harmonized framework [19]
Romania, 2024–2025	*Campylobacter* spp.; slaughterhouse and retail poultry meat	41.6%	2.8%	25.0%	Kirby–Bauer disc diffusion; food-derived isolates [20]
China, 2020	*Campylobacter* spp.; chicken- and pig-origin isolates	64.5%	75.3%	68.8%	Broth microdilution; farm-derived isolates [21]

Abbreviations: CLSI, Clinical and Laboratory Standards Institute; EU, European Union; EUCAST, European Committee on Antimicrobial Susceptibility Testing; EURL, European Union Reference Laboratory; WHONET, WHO-supported antimicrobial resistance surveillance software.

**Table 2:** *Campylobacter* virulence factors by disease stage, mechanistic evidence, and supporting model.

Factor	Disease Stage	Mechanistic Evidence	Interpretation/Natural-Disease Relevance	Species/Principal Model
Flagellar motility (FlaA)	Colonization	Strong mechanistic	Loss of motility impairs chick colonization; *flaA* also contributes to epithelial invasion in vitro [24,25,26]. No inference to clinical severity.	*C. jejuni*; chick colonization plus epithelial-cell assays
Chemotaxis (Tlp/Che)	Colonization	Strong for colonization	Mutant studies support a colonization role; direct relevance to human disease severity is uncertain [5,6].	Predominantly *C. jejuni*; mutant colonization work
N-/O-glycosylation (*pgl*)	Colonization/immune	Moderate mechanistic	*pgl* mutants impair attachment and chick colonization [27]; the contribution to natural human disease has not been quantified.	*C. jejuni*; epithelial-cell and chick experiments
CadF, FlpA adhesins	Epithelial attachment	Strong mechanistic	Attachment and colonization are supported in vitro and in vivo; CadF function has been demonstrated in both species [28,29].	*C. jejuni* dominant; CadF function also shown in *C. coli*; cell and chicken models
Cia proteins, fT3SS	Invasion	Moderate; mainly in vitro	*ciaB* disruption lowers Cia secretion and invasion; most evidence comes from cell culture [30,31].	*C. jejuni*; chiefly cell-culture evidence
HtrA protease	Translocation	Moderate; mainly in vitro	Junction cleavage and translocation are reproducible experimentally, but the natural-disease contribution is unresolved [32].	*C. jejuni*; epithelial monolayer models
CDT (CdtA/B/C)	Host damage	Strong mechanistic	CdtB DNase activity and G2/M arrest are established; the effect on natural disease severity is less certain [33,34].	Mostly *C. jejuni*; cellular toxin and vesicle studies
Capsule	Persistence	Moderate mechanistic	Phase-variable capsule supports serum resistance and surface variation [1]; its clinical contribution is not quantified.	Mostly *C. jejuni*; serum-resistance and surface-variation studies
Biofilm/quorum sensing (QS; AI-2)	Transmission	Context-dependent; mainly in vitro	Evidence is mainly in vitro, and *luxS* phenotypes may include metabolic effects; direct human-disease causality is unproven [35].	Mostly *C. jejuni*; in vitro biofilm/QS work
Type VI secretion system	Colonization/competition	Context-dependent	Restricted to a subset of isolates; prevalence and phenotype vary by strain, source, and assay [36].	Subset of isolates; strain-dependent models
Phase variation/contingency loci	Immune evasion and colonization	Moderate/context-dependent	Slipped-strand mispairing changes LOS, capsule, and flagellar glycosylation, generating reversible surface heterogeneity [1].	*C. jejuni*-heavy evidence; genomic and phenotypic studies
Stress survival (aerotolerance, viable but nonculturable [VBNC])	Persistence/transmission	Context-dependent	Aerotolerance, oxidative-stress responses, and VBNC states aid food-chain persistence; the effect on disease is indirect [37,38,39].	Mostly *C. jejuni*; food/environmental stress models
LOS ganglioside mimicry	Post-infectious (GBS)	Strong mechanistic link; low penetrance	Mechanism strong but penetrance low; GBS follows roughly 0.2–1.7 per 1000 illnesses and varies with host and strain [15,16,40].	*C. jejuni*; animal models plus human epidemiology

**Table 3:** Evidence linking major resistance determinants with fitness-, colonization-, or virulence-related phenotypes in *Campylobacter.*

Determinant/Observation	Reported Phenotype	Evidence Type/Model	Evidence Strength	Main Limitation
*gyrA* Thr-86-Ile	Greater in-host competitiveness in chickens	Isogenic or clonally related mutants; in vivo competition	Strong for fitness	Background-dependent; no evidence of greater clinical virulence [51]
*gyrA* Thr-86-Ile	DNA supercoiling and pleiotropic transcriptional effects	Mechanistic; in vitro	Moderate	Natural-disease consequence not directly tested [52]
Fluoroquinolone-resistant phenotype	More biofilm formation or altered host–cell phenotypes in some strains	In vitro/cell culture	Moderate in vitro	Strain-specific; no multi-background isogenic in vivo validation [53]
CmeABC	Bile resistance and intestinal colonization	Mutant and in vivo studies	Strong for fitness	Conserved colonization system rather than an acquired resistance determinant [58,59]
*erm(B)*/aminoglycoside determinants in MDRGIs	Co-selection of linked resistance cassettes; possible linked traits	Genomic co-location	Weak/associational	Co-location alone does not establish a fitness effect [55,56]
Resistant clonal complexes	Virulence-associated phenotypes enriched in resistant lineages	Population/epidemiological association	Weak/confounded	Lineage-level co-inheritance can mimic causation [4,51]

## Data Availability

No new data were created or analyzed in this study. Data sharing is not applicable to this review.

## Abbreviations

The following abbreviations are used in this manuscript:

WHO	World Health Organization
LOS	Lipooligosaccharide
GBS	Guillain-Barré syndrome
IBS	Irritable bowel syndrome
CDT	Cytolethal distending toxin
T3SS	Type III secretion system
T6SS	Type VI secretion system
MDR	Multidrug resistance
MDRGI	Multidrug resistance genomic island(s)
RE-CmeABC	Resistance-enhancing CmeABC
AI-2	Autoinducer-2
fT3SS	Flagellar type III secretion system
AMR	Antimicrobial resistance
QS	Quorum sensing
VBNC	Viable but nonculturable
